# A High-Efficiency Wireless Information and Energy Co-Transmission System Based on Self-Compensating Inductive Temperature Sensitivity Error

**DOI:** 10.3390/s25082459

**Published:** 2025-04-14

**Authors:** Tan Lu, Libo Ding, Keren Dai, Shaojie Ma, He Zhang

**Affiliations:** School of Mechanical Engineering, Nanjing University of Science and Technology, Nanjing 210094, China; soilegg@njust.edu.cn (T.L.); dinglibo@njust.edu.cn (L.D.); dkr@njust.edu.cn (K.D.); shaojiem@njust.edu.cn (S.M.)

**Keywords:** wireless power and information transfer system, resonant frequency, temperature compensation, Direct Digital Synthesis (DDS)

## Abstract

To address the stability issues of energy and information transmission in wireless power and information transfer system operating over a wide temperature range, this paper establishes a mathematical model of the resonant frequency of an electromagnetic coupling system under varying temperature conditions. Simulations and experiments are conducted to analyze the impact of temperature on resonance characteristics. The results show that within the temperature range of −40 °C to 50 °C, frequency deviation leads to a reduction in the power deviation coefficient to 35.93%. To mitigate this issue, a real-time frequency compensation method based on Direct Digital Synthesis (DDS) is proposed, which dynamically adjusts the operating frequency to ensure that the system remains in optimal resonance. The experimental results demonstrate that the proposed method reduces the system’s operating frequency error from 3 kHz to within 0.2 kHz (a 93.33% reduction), restoring the power deviation coefficient to 0.54% and significantly improving system stability and reliability. This study provides theoretical support and engineering insights for the optimization of electromagnetic coupling wireless power and the information transfer system under wide temperature conditions.

## 1. Introduction

### 1.1. Electromagnetic Coupling-Based Wireless Power and Information Transfer Technology

The wireless energy and information simultaneous transmission system is an advanced technology that enables the concurrent transfer of energy and data through electromagnetic coupling or electromagnetic waves. This technology has found widespread applications in wireless charging, smart devices, industrial automation, and military equipment. At its core, the system differentiates energy transfer from signal transmission by utilizing distinct frequency bands or modulation techniques, ensuring efficient and interference-free operation. Key advantages of this approach include the elimination of mechanical contacts, high-speed and high-frequency performance, and strong resistance to external disturbances, making it highly suitable for demanding and complex environments. However, the stability of the transfer system is influenced by multiple factors, such as environmental temperature fluctuations, magnetic coupling characteristics, and the limited operational time window. In particular, under extreme temperature conditions ranging from −40 °C to 50 °C, the system’s resonant frequency tends to drift, leading to detuning, which in turn compromises the efficiency and reliability of both energy and data transmission. Therefore, investigating the impact of temperature variations on the resonant system and developing an effective frequency compensation method is essential for enhancing the stability and adaptability of the wireless power and information transfer system.

In a wireless power and information transfer system, the stability of the resonant circuit is a key factor affecting power deviation coefficient. In practical applications, system performance is influenced by multiple physical factors, including secondary coil load variations, primary-secondary coil alignment, and environmental temperature [1,2]. To achieve a stable coupling between the receiving device and the transfer system, these factors must be comprehensively considered. Existing studies have primarily focused on resonant frequency drift and long-distance coupling efficiency degradation. For example, the impact of load switching on resonant frequency has been analyzed, and primary-side frequency tracking control has been introduced to restore resonance rapidly, thereby enhancing system stability [3]. An adaptive frequency-tracking tuning method has been proposed to mitigate power attenuation in the 5–20 cm transmission range, maintaining wireless power transfer efficiency above 80% [4]. Additionally, temperature-induced inductance variations have been studied, demonstrating that a 10 μH planar PCB coil operating at 50 kHz exhibits inductance fluctuations of approximately 0.05 μH, validating the influence of temperature on inductance characteristics [5]. However, these studies primarily focus on ambient or high-temperature conditions, while low-temperature inductance variations and their impact on resonant characteristics remain insufficiently explored. Given that the transfer system must operate reliably between −40 °C and 50 °C, further research on detuning effects in low-temperature environments and their compensation methods is urgently needed.

Electromagnetic coupling-based wireless power and information transfer technology has demonstrated significant advantages in improving system stability [6]. For instance, optimized coil design and control strategies have been shown to significantly enhance system stability and reliability [7,8,9]. Studies on performance variations under different environmental conditions indicate that well-designed system architectures and control strategies can effectively improve adaptability and operational robustness [10,11]. Under extreme temperature conditions, temperature compensation-based control strategies have been proposed to enhance system stability and reliability [12]. Research on transmission characteristics at different frequencies suggests that optimizing the operating frequency can significantly improve transmission efficiency and system stability [13]. Moreover, a multi-frequency coupling-based wireless power and information transfer system has been introduced, where the simultaneous transmission of energy and data at multiple frequencies significantly enhances interference resistance [14]. Further studies have shown that appropriate load matching and frequency adjustment can effectively boost transmission efficiency and stability [15]. The application of adaptive frequency-tracking control strategies across varying coupling distances has also demonstrated substantial improvements in energy transfer efficiency [16]. Additionally, studies on different magnetic field intensities have revealed that optimizing field strength and coil design can significantly enhance system efficiency and stability [17]. Control strategies based on magnetic field intensity feedback, enabling real-time monitoring and system parameter adjustments, have been proven effective in improving operational stability and reliability [18]. Temperature compensation and frequency adjustment strategies have been validated as key methods for maintaining high transmission efficiency and stability under varying thermal conditions [19]. Finally, an integrated approach combining temperature compensation and frequency-tracking control has been shown to further enhance system robustness under extreme environmental conditions [20,21].

### 1.2. Resonant Circuit Model of the Wireless Power and Information Transfer System

The electromagnetic coupling-based wireless power and information transfer system operates on the principle of magnetic resonance coupling, enabling efficient, contactless energy, and data transfer between the transmission setter (primary circuit) and receiving device (secondary circuit). This system must complete data synchronization within an extremely short time window (15 ms) to ensure precise reliable working. The core mechanism of the system lies in maintaining resonant conditions, where optimal power transfer is achieved by ensuring that the system operates at its resonant frequency.

**Figure 1:**
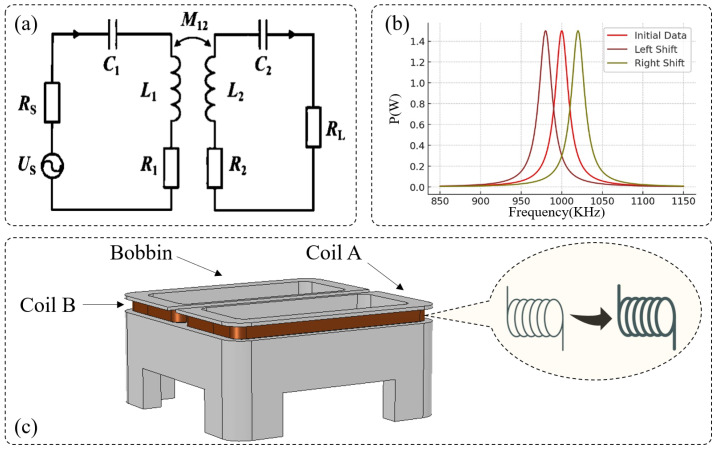
(**a**) Equivalent circuit of the resonant system. (**b**) Relationship between the output power of the resonant system and the operating frequency. (**c**) Inductance variation.

As illustrated in Figure 1a, the basic circuit structure of the wireless power and information transfer system consists of a primary resonant circuit (comprising a capacitor $C_{1}$, an inductor $L_{1}$, and a resistance $R_{s}$) and a secondary resonant circuit (comprising a capacitor $C_{2}$ and an inductor $L_{2}$). The primary coil $L_{1}$ is constructed using multiple turns of copper wire and, together with capacitor $C_{1}$, forms an LC resonant circuit. When the drive circuit applies a high-frequency alternating current (AC) signal, $L_{1}$ generates an alternating magnetic field, inducing energy transfer to the secondary coil $L_{2}$ through magnetic resonance coupling. The secondary coil $L_{2}$ receives the transmitted energy via magnetic induction and forms resonance with its matching capacitor $C_{2}$, ensuring maximum power transfer to the receiving device. This efficient energy and data transmission mechanism allows the receiving device to be set without physical contact, providing high-speed operation, enhanced durability, and strong resistance to environmental interference.

This energy and information simultaneous transmission system is also capable of completing the transmission of information. The communication data are modulated onto the energy carrier, enabling the simultaneous transmission of information and energy during the coupling process between the primary and secondary coils. The modulation technique employed is amplitude modulation. Each data unit consists of seven bytes, equivalent to 56 bits of binary coding, which includes both the frame header and checksum. The encoding waveforms for binary digits ‘0’ and ‘1’ are each 200 microseconds in duration, with differing high-to-low level ratios, resulting in a comprehensive communication rate of 5 kbit/s. In the control and drive circuits, the transmission setter receives hexadecimal information, performs modulation, and drives the transmission process. During the coupling process of the primary and secondary coils, data are transmitted in the form of fixed waveforms encoded in binary code. Upon reception, the secondary coil forwards the data to a subsequent processing chip for decoding and storage.

### 1.3. Mathematical Model of the Resonant System

The resonant frequency of the wireless power and information transfer system is determined by the LC oscillation principle, which can be expressed as:

(1) $$f_{0}=\frac{1}{2\pi \sqrt{L_{1}C_{1}}}$$

where $L_{1}$ is the inductance of the primary coil, and $C_{1}$ is the matching capacitor. To ensure optimal energy transfer efficiency, the operating frequency of the transmission setter $f_{1}$ must match the system’s resonant frequency $f_{0}$, satisfying the condition:

(2) $$f_{1}=f_{0}$$

When the operating frequency $f_{1}$ deviates from the resonant frequency $f_{0}$, the power transfer efficiency drops significantly. Simulation analysis shows that at the optimal resonant point ($f_{1}$ = $f_{0}$ = 1 MHz), the wireless power and information transfer system achieves a maximum output power of 1.6667 W. However, if $f_{1}$ shifts away from $f_{0}$, the transmitted power declines sharply, impairing the effectiveness of both energy and data transmission. As shown in Figure 1b, the power transmission efficiency decreases as the deviation between $f_{1}$ and $f_{0}$ increases. Under extreme temperature conditions (e.g., −40 °C), changes in $L_{1}$ and $C_{1}$ due to temperature variations cause $f_{0}$ to shift. As a result, the mismatch between $f_{1}$ and $f_{0}$ increases, leading to a drop in output power to 1.0678 W, which is only 64.07% of the maximum power. This phenomenon highlights the importance of maintaining frequency matching under varying environmental temperatures, as a failure to do so may result in unstable data synchronization and reduced energy transfer efficiency.

To ensure the reliability and efficiency of the wireless power and information transfer system, it is necessary to dynamically adjust the operating frequency $f_{1}$ in response to temperature variations, ensuring continuous synchronization with the temperature-dependent resonant frequency $f_{0}$. The following sections introduce a real-time frequency compensation method based on Direct Digital Synthesis (DDS) technology, which dynamically tunes $f_{1}$ to improve resonance matching, thereby enhancing the stability and efficiency of both energy and data transmission.

Direct Digital Synthesis (DDS) offers advantages such as high-frequency resolution, rapid tuning capability, and low power consumption, making it well-suited for dynamic frequency adjustment in wireless power and information transfer systems to ensure optimal resonance matching. Research has demonstrated that DDS exhibits significant advantages in frequency compensation, effectively enhancing system stability and adaptability through real-time frequency adjustments [22]. Its superior frequency resolution and tuning speed make it ideal for high-precision frequency control [23], and its integration with temperature compensation strategies has been shown to significantly enhance system stability and reliability under extreme environmental conditions [24]. In high-frequency applications, DDS exhibits even greater performance advantages, making it particularly effective for precision control [25]. Additionally, DDS-based multi-frequency coupling systems enable simultaneous energy and data transmission, significantly enhancing interference resistance and system stability [26]. Furthermore, system efficiency can be optimized through load matching and frequency adjustments [27]. Adaptive frequency-tracking strategies have also been proven effective in improving transmission efficiency and stability across varying coupling distances [28,29]. To address the impact of extreme temperature variations on wireless power and information transfer systems, this paper proposes a real-time frequency compensation method based on Direct Digital Synthesis (DDS). This approach dynamically adjusts the operating frequency to counteract temperature-induced resonance frequency drift, thereby ensuring stable and efficient system performance across a wide temperature range.

## 2. Resonant Frequency Temperature Drift Characteristics

### 2.1. Impact of Frequency Drift on Output Power

To analyze the effect of resonant frequency drift on system output power, the calculated resonant frequencies $f_{0}$ at different temperatures were substituted along with a fixed operating frequency $f_{1}$ (1 MHz) into the power-frequency response curve shown in Figure 1b. The corresponding output power values were determined. The simulation results indicate that when the temperature deviates from 20 °C, the output power of the resonant system decreases significantly. Moreover, as the temperature deviation increases, power loss becomes more severe. In particular, at −40 °C, the system exhibits the lowest output power of 1.0678 W, which corresponds to only 64.07% of the maximum power output. Under this condition, the system is in a severely detuned state, making it incapable of ensuring the proper power and information transmission. In summary, environmental temperature variations substantially impact the resonant frequency $f_{0}$, leading to an increased frequency mismatch between $f_{0}$ and the operating frequency $f_{1}$. This mismatch results in reduced energy transfer efficiency and compromised system performance. To mitigate these effects, a real-time dynamic compensation mechanism is required to continuously adjust the operating frequency $f_{1}$, thereby compensating for temperature-induced frequency drift and ensuring the stable operation of the wireless power and information transfer system under varying temperature conditions.

### 2.2. Influence of Temperature on Resonant Frequency

To investigate the effect of temperature variations on the resonant frequency $f_{0}$, the simulated values of inductance $L_{1}$ and capacitance $C_{1}$ over the temperature range of −40 °C to 50 °C were substituted into the resonant frequency formula. The calculated values of $f_{0}$ at different temperatures are presented in Table 1.

The simulation results indicate that within the temperature range of −40 °C to 50 °C, the resonant frequency $f_{0}$ decreases as the temperature increases. At 20 °C, the system operates at its optimal resonance condition, where the operating frequency $f_{1}$ matches the resonant frequency $f_{0}$ at 1 MHz, achieving maximum power output of 1.6667 W. However, when the temperature increases to 50 °C, the resonant frequency drops to 0.99752 MHz, and when the temperature decreases to −40 °C, the resonant frequency increases to 1.00352 MHz.

These temperature-induced frequency shifts highlight the necessity of real-time frequency compensation, ensuring that the operating frequency $f_{1}$ dynamically adjusts to match the changing resonant frequency $f_{0}$. Such an approach is crucial for maintaining optimal energy transfer efficiency and stable data transmission across a wide temperature range.

### 2.3. Resonant Frequency Calculation Model

The resonant frequency $f_{0}$ of the system is determined by the actual values of the inductance $L_{1}$ and the capacitance $C_{1}$. For the resonant system studied in this research, the design resonant frequency at an ideal temperature of 20 °C is 1 MHz. However, under varying temperature conditions, $L_{1}$ is significantly affected by temperature fluctuations, while $C_{1}$, which is an NP0 surface-mount capacitor (temperature coefficient 0 ± 30 ppm/°C), remains relatively stable. Consequently, inductance variation is the primary factor influencing resonant frequency drift. In practical operation, the operating frequency $f_{1}$ of the wireless power and information transfer system must remain synchronized with the resonant frequency $f_{0}$ to ensure optimal resonance conditions for efficient energy and data transmission.

## 3. Inductance Temperature Characteristic Simulation

### 3.1. Structure of the Transmission Setter

The transmission setter, as the core component of the resonant system, is directly connected to the driving circuit and is responsible for transmitting energy and data to the receiving device, ensuring precise transmission. The physical structure of the transmission setter adopts a multi-layer stacked coil design, where 0.19 mm diameter copper-core enameled wire is tightly wound around a polyoxymethylene (POM) frame, forming a highly efficient electromagnetic coupling structure. The overall dimensions of the coil are 29 mm × 12.5 mm, with coil A and coil B each consisting of 10 turns wound in opposite directions to enhance the magnetic field intensity. The transmission setter applies an input signal to the primary coil, which generates a stable alternating magnetic field based on the principle of magnetic coupling, thereby enabling non-contact energy and data transfer to the receiving device.

### 3.2. Electromagnetic Simulation Model Development

The resonant characteristics of the wireless power and information transfer system are significantly influenced by the inductance of the primary coil $L_{1}$, which varies under different temperature conditions. To analyze the effect of temperature on the resonant system, an electromagnetic simulation model was developed using COMSOL Multiphysics 6.2, allowing for the precise computation of temperature-dependent inductance variations.

In COMSOL, a three-dimensional model of the transmission setter was constructed, as shown in Figure 1c. The electromagnetic field was modeled based on Maxwell’s equations, expressed as:

(3) $$▽*H=J+\frac{\partial d}{\partial t}$$

where *H* represents magnetic field intensity, *J* is current density, and *D* is the electric displacement vector.

**Figure 2:**
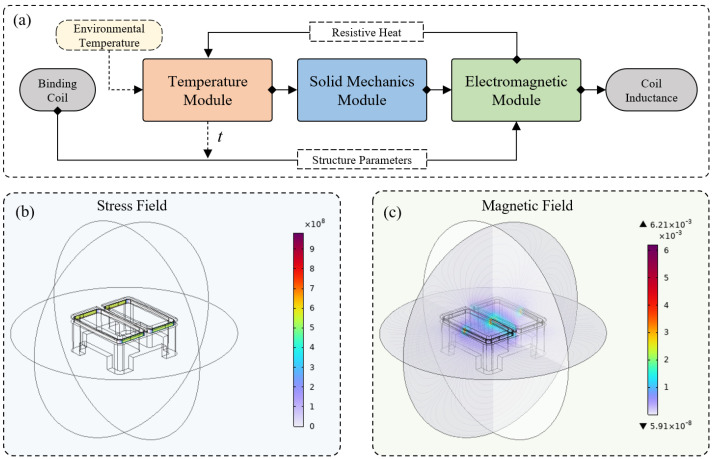
(**a**) Multi-physics coupling process. (**b**) Stress field under −20 °C. (**c**) Magnetic field under −20 °C.

Since temperature variations not only affect the magnetic properties of the coils but also lead to thermal expansion effects of the materials, a solid mechanics field was incorporated to simulate temperature-induced structural changes in the transmission setter. The thermal expansion coefficients for copper ($\alpha_{1}$) and polyoxymethylene (POM) ($\alpha_{2}$) were defined accordingly. Additionally, considering the self-heating effect of the transmission setter coil during prolonged operation, a solid heat transfer field was established to define the material’s thermal conductivity properties. These fields were coupled with the electromagnetic field and solid mechanics field, forming a comprehensive multi-physics simulation model. Finally, through high-precision mesh refinement and numerical simulations, the inductance variation trend of $L_{1}$ under different temperature conditions was obtained, as illustrated in Figure 2a.

### 3.3. Relationship Between Inductance $L_{1}$ and Temperature t

In the developed simulation model, the operating temperature t was set within a range of −40 °C to 50 °C, with 10 °C intervals, resulting in a total of 10 temperature data points. The inductance value $L_{1}$ of the resonant system was calculated at each temperature point, and a temperature-inductance relationship model was established using curve fitting:

(4) $$L_{1}=1.22\;\times \;10^{-8}t^{3}+5.61\;\times \;10^{-6}t^{2}+0.00155t+15.80291$$

**Figure 3:**
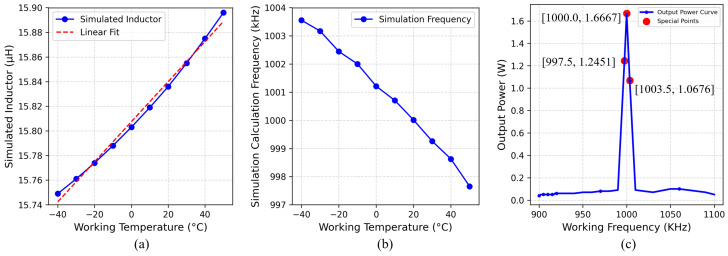
(**a**) Inductance value. (**b**) Resonance frequency value. (**c**) System power value over a wide temperature range.

The fitting accuracy was 0.99999, indicating a high level of computational precision. The simulation results, as illustrated in Figure 3a, demonstrate that $L_{1}$ increases with rising operating temperature. Specifically, at 50 °C, the inductance increased by 0.147 μH compared to its value at −40 °C.

### 3.4. Relationship Between Inductance $L_{1}$ and Operating Time

Due to resistive heating effects, the transmission setter coil experiences self-heating during continuous operation, which may cause variations in its inductance $L_{1}$. To analyze this effect, a solid heat transfer field was incorporated into the simulation model, defining both the heat source and thermal conductivity properties. The operating current was set to 1A, and the temperature variation of the transmission setter coil was simulated over a 10-min continuous operation period.

The simulation results indicate that within 10 min, the coil temperature gradually increased, reaching a final rise of 1.2 °C. However, this minor temperature increase had negligible effects on $L_{1}$, leading to insignificant inductance variations that do not compromise the stability of the resonant system. Considering the heat exchange between the transmission setter and the external environment, the self-heating effect during prolonged operation can be neglected in terms of its influence on $L_{1}$. Therefore, the inductance of the transmission setter is primarily influenced by environmental temperature variations, while long-term operation itself does not significantly affect its resonant characteristics.

## 4. DDS Real-Time Frequency Compensation Method

### 4.1. Overview of DDS Compensation Technology

To ensure that the wireless power and information transfer system maintains resonance under varying temperature conditions, this study employs Direct Digital Synthesis (DDS) technology to achieve real-time frequency compensation for the operating frequency of the transmission setter. DDS technology offers high-frequency resolution, rapid tuning capability, low cost, and low power consumption, allowing for dynamic frequency adjustments based on environmental temperature changes to maintain system stability. The specific design parameters are as follows: sinusoidal table capacity is 1024 points, and each point uses 2 bytes to store the phase DAC data. The DAC is 12-bit, and the update rate is 10 MHz. Both the phase accumulator and the frequency control word are realized with 32-bit integer variables. Take the first 10 bits of the phase accumulator to look up the table and output, and adjust the frequency control word according to the temperature to realize the frequency control of the output signal, thereby enabling efficient real-time frequency compensation.

### 4.2. Frequency Compensation Model

Based on the temperature–frequency relationship curve presented in Table 1, a third-order curve fitting was applied to the experimental data, yielding the temperature–frequency compensation equation:

(5) $$\Delta f=3.568375\;\times \;10^{-10}t^{3}+1.728603\;\times \;10^{-7}t^{2}+6.422188\;\times \;10^{-5}t-1.202414\;\times \;10^{-3}$$

where:

- Δf is the required frequency compensation value (MHz);
- t is the current ambient temperature (°C);

The correlation coefficient of the fitted equation is 0.99992, indicating high accuracy in describing the effect of temperature variation on the resonant system.

During system operation, the microcontroller continuously monitors the ambient temperature using a temperature sensor. The computed compensation value Δf is then applied by the DDS frequency signal source, which dynamically adjusts the output frequency to ensure that the operating frequency $f_{1}$ remains synchronized with the resonant frequency $f_{0}$ at the current temperature. This approach effectively prevents frequency drift caused by temperature fluctuations, thereby enhancing system stability and performance.

### 4.3. Experimental Validation

#### 4.3.1. Experimental Setup

- The model of the Oscilloscope is DSOX4024A of keysight Company, 200 MHz, and boasts a 1-million-waveforms/second update rate.
- The high–low temperature test chamberis produced by Huasheng Company, and its detailed parameters are as follows: the temperature ranges from −50 °C to 150 °C, the temperature fluctuation is ±0.5 °C, the temperature uniformity is ±2 °C, and the temperature gradient is 5 °C/min.

#### 4.3.2. Coil Inductance Variation Test

**Figure 4:**
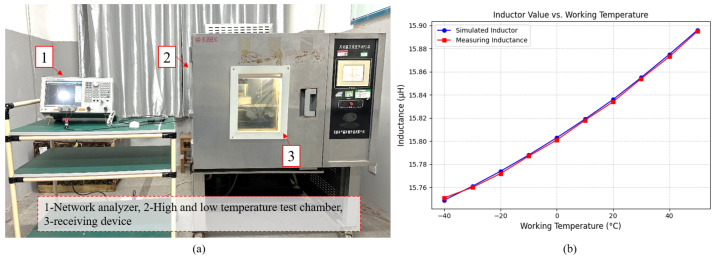
(**a**) Inductance measurement test equipment. (**b**) Relationship between simulated inductance and measured inductance.

To verify the effect of environmental temperature on coil inductance $L_{1}$, an experiment was conducted using six standard receiving devices, measuring their inductance values under different temperature conditions. The experimental setup, as shown in Figure 4a, consisted of the following key components:

- A vector network analyzer (for precise inductance measurement),
- A high–low temperature test chamber (to simulate environmental temperature variations),
- A transmission setter (to replicate actual operating conditions).

The experiment covered a temperature range of −40 °C to 50 °C, with measurements taken at 10 °C intervals. The measured inductance values were then compared with the simulated values, as presented in Table 2.

The results indicate that the measured inductance values closely align with the simulated values, demonstrating a high degree of consistency. Additionally, the data confirm that $L_{1}$ increases with rising temperature, further validating the influence of temperature variations on the resonant characteristics of the wireless power and information transfer system. These findings reinforce the necessity of real-time frequency compensation, as temperature-induced inductance variations directly affect the resonant frequency, leading to potential detuning if left uncorrected.

#### 4.3.3. Frequency Compensation Experiment

To verify the effectiveness of the DDS frequency compensation method in optimizing the resonance matching of the wireless power and information transfer system under different temperature conditions, a series of experimental tests were designed and conducted. The primary focus of these experiments was to analyze the operating frequency error and power deviation coefficient, evaluating the performance variations before and after compensation.

**Figure 5:**
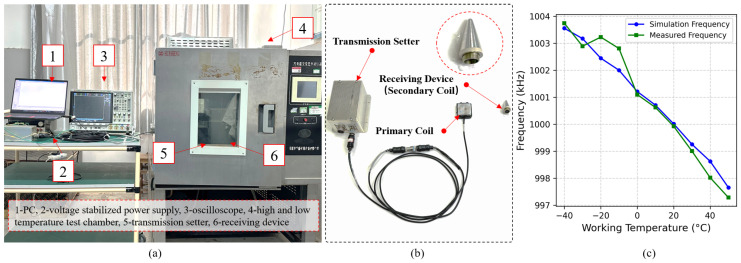
(**a**) System test equipment. (**b**) Wireless power and information transfer system. (**c**) Relationship between simulated resonant frequency and measured frequency.

The experimental platform, as illustrated in Figure 5a, consists of the following key components:

- The wireless power and information transfer system: includes the transmission setter (primary coil) and receiving device (secondary coil), simulating actual working conditions.
- The DDS Frequency Control Unit: Utilizes a 32-bit microcontroller (MCU) to control the DDS frequency synthesizer, dynamically adjusting the operating frequency based on temperature variations to ensure optimal resonance.
- The Temperature Control System: Employs a high–low temperature test chamber (temperature range: −40 °C to 50 °C, accuracy: ±0.1 °C) to simulate extreme environmental conditions.
- The Testing Equipment: Includes a digital oscilloscope (for measuring operating frequency), a vector network analyzer (for monitoring resonance characteristics), and a regulated power supply (for providing stable input voltage).
- The Data Acquisition and Analysis System: Records temperature variations, frequency error, and power changes in real time, enabling precise performance assessment.

This comprehensive experimental setup ensures the accurate evaluation of the DDS frequency compensation method, allowing for a detailed comparison of system behavior before and after frequency compensation across a wide temperature range.

#### 4.3.4. Experimental Procedure and Results

The experiment employed a stepwise temperature ramping method, covering a temperature range of −40 °C to 50 °C with 10 °C increments. The procedure consisted of the following steps:

- Temperature Stabilization: The wireless power and information transfer system was placed inside a high–low temperature test chamber and maintained at each target temperature for 10 min to ensure thermal equilibrium.
- Frequency Measurement: At each temperature point, the operating frequency $f_{1}$ of the wireless power and information transfer system was recorded before and after DDS compensation. The relative frequency error was computed as:
  (6) $$\Delta f=\left|\frac{f_{1}-f_{0}}{f_{0}}\right|*100\%$$
- Power and Efficiency Measurement: let $P_{0}$ be the theoretical resonant power. The system’s working power $P_{1}$ was measured under the same conditions, and the power deviation coefficient $\delta_{P}$ was calculated using:
  (7) $$\delta_{P}=1-\frac{P_{1}}{P_{0}}$$
  to evaluate the effectiveness of the compensation strategy.

#### 4.3.5. Analysis of Results

As shown in Table 3, before DDS frequency compensation, the operating frequency $f_{1}$ deviated significantly from the ideal resonant frequency $f_{0}$, with the deviation increasing as temperature varied. The highest frequency error occurred at −40 °C, reaching 0.352%, which resulted in a reduction in power deviation coefficient to 35.93%. Although the error decreased as temperature increased, at 50 °C, a deviation of −0.248% still posed a risk to system stability.

After implementing DDS frequency compensation, the wireless power and information transfer system was able to dynamically adjust the operating frequency, ensuring that $f_{1}$ remained synchronized with $f_{0}$ under all temperature conditions. Experimental data confirmed that after compensation, the frequency error at all temperature points was reduced to within 0.2 kHz. This effectively eliminated the impact of temperature drift. Additionally, the power deviation coefficient significantly improved, with all temperature point power deviation coefficients below 0.54%. These results demonstrate that DDS-based real-time frequency compensation enables the wireless power and information transfer system to maintain stable operation even under extreme environmental conditions, ensuring high-precision energy and data transfer.

## 5. Conclusions

This study addresses the resonant frequency drift issue in electromagnetic coupling-based wireless power and information transfer systems by proposing a real-time frequency compensation method based on Direct Digital Synthesis (DDS). The proposed method utilizes temperature monitoring and dynamic frequency adjustment to maintain resonance matching across varying temperature conditions, ensuring stable energy and data transmission. A mathematical model of the resonant system was established, analyzing the influence of temperature on inductance and resonant characteristics. Based on this, a DDS-based frequency compensation strategy was developed, where a microcontroller dynamically adjusts the operating frequency in real time, enhancing system stability and adaptability. Experimental validation confirmed that this method effectively mitigates temperature-induced frequency drift, significantly improving the reliability of the wireless power and information transfer system. Compared with conventional methods, DDS frequency compensation offers high precision, rapid response, and low power consumption, making it well-suited for complex operational environments. Furthermore, this approach can be extended to wireless power and data transmission applications.

Future research may explore the integration of intelligent algorithms to further optimize compensation strategies, enhancing system accuracy and energy efficiency to meet a broader range of engineering applications.

## Figures and Tables

**Table 1:** Relationship between resonant frequency $f_{0}$ and operating temperature.

Operating Temperature (°C)	Inductance $L_{1}$ (μH)	Capacitance $C_{1}$ (nF)	Resonant Frequency $f_{0}$ (MHz)
−40	15.749	1.5971	1.00352
−30	15.761	1.5976	1.00298
−20	15.774	1.5981	1.00242
−10	15.788	1.5986	1.00183
0	15.803	1.5990	1.00120
10	15.819	1.5995	1.00054
20	15.836	1.6000	0.99986
30	15.855	1.6005	0.99911
40	15.875	1.6010	0.99833
50	15.896	1.6014	0.99752

**Table 2:** Coil inductance measurement results.

Operating Temperature (°C)	Simulated Inductance $L_{1}$ (μH)	Measured Inductance $L_{1}$ (μH)
−40	15.749	15.751
−30	15.761	15.760
−20	15.774	15.772
−10	15.788	15.787
0	15.803	15.801
10	15.819	15.818
20	15.836	15.834
30	15.855	15.854
40	15.875	15.873
50	15.896	15.895

**Table 3:** Power deviation coefficient before and after DDS compensation.

Operating Temperature (°C)	Output Power Before Compensation (*W*)	$\delta_{P}$ Before Compensation (%)	$\delta_{P}$ After Compensation (%)
−40	1.06	36.53	0.25
−30	1.17	29.94	0.54
−20	1.28	23.35	0.03
−10	1.41	15.57	0.07
0	1.54	7.78	0.15
10	1.64	1.8	0.06
20	1.67	0.0	0.0
30	1.58	5.39	0.12
40	1.43	14.37	0.07
50	1.25	25.15	0.08

## Data Availability

Data is contained within the article.

## Abbreviations

The following abbreviations are used in this manuscript:

DDS	Direct Digital Synthesis
MCU	Microcontroller Unit
POM	Polyoxymethylene
